# Multiple Instance Learning for the Detection of Lymph Node and Omental Metastases in Carcinoma of the Ovaries, Fallopian Tubes and Peritoneum

**DOI:** 10.3390/cancers17111789

**Published:** 2025-05-27

**Authors:** Katie E. Allen, Jack Breen, Geoff Hall, Georgia Mappa, Kieran Zucker, Nishant Ravikumar, Nicolas M. Orsi

**Affiliations:** 1 Leeds Institute of Medical Research, University of Leeds, Leeds LS9 7TF, UKn.m.orsi@leeds.ac.uk (N.M.O.); 2 School of Computing, University of Leeds, Leeds LS2 9JT, UK; 3 Leeds Cancer Centre, University of Leeds, Leeds LS9 7TF, UK

**Keywords:** ovarian carcinoma, metastasis detection, digital pathology, computational pathology, computer vision

## Abstract

**Background/Objectives**: Surgical pathology of tubo-ovarian and peritoneal cancer carries a well-recognised diagnostic workload, partly due to the large amount of non-primary tumour-related tissue requiring assessment for the presence of metastatic disease. The lymph nodes and omentum are almost universally included in such resection cases and contribute considerably to this burden, principally due to volume rather than task complexity. To date, artificial intelligence (AI)-based studies have reported good success rates in identifying nodal spread in other malignancies, but the development of such time-saving assistive digital solutions has been neglected in ovarian cancer. This study aimed to detect the presence or absence of metastatic ovarian carcinoma in the lymph nodes and omentum. **Methods**: We used attention-based multiple-instance learning (ABMIL) with a vision-transformer foundation model to classify whole-slide images (WSIs) as either containing ovarian carcinoma metastases or not. Training and validation were conducted with a total of 855 WSIs of surgical resection specimens collected from 404 patients at Leeds Teaching Hospitals NHS Trust. **Results**: Ensembled classification from hold-out testing reached an AUROC of 0.998 (0.985–1.0) and a balanced accuracy of 100% (100.0–100.0%) in the lymph node set, and an AUROC of 0.963 (0.911–0.999) and a balanced accuracy of 98.0% (94.8–100.0%) in the omentum set. **Conclusions**: This model shows great potential in the identification of ovarian carcinoma nodal and omental metastases, and could provide clinical utility through its ability to pre-screen WSIs prior to histopathologist review. In turn, this could offer significant time-saving benefits and streamline clinical diagnostic workflows, helping to address the chronic staffing shortages in histopathology.

## Simple Summary

Ovarian cancer staging hinges on the histopathological evaluation of large amounts of non-primary tumour-related tissue (e.g., lymph nodes and omentum) for the presence of metastatic disease. This study aimed to determine whether artificial intelligence could effectively identify nodal and omental metastatic cancer deposits using attention-based multiple-instance learning to classify whole-slide images (WSIs) as either containing tumour cells or not. Training and validation were conducted with a total of 855 WSIs of surgical specimens from 404 patients. All objective measures of accuracy demonstrated the model’s great potential in identifying metastatic disease. In the clinical setting, this model could potentially pre-screen WSIs prior to histopathologist review, offering significant time-saving benefits and streamlining clinical diagnostic workflows.

## 1. Introduction

Tubo-ovarian and peritoneal cancer is the eighth most prevalent cancer among women globally, and is often associated with a poor prognosis, with 324,000 new diagnoses leading to 206,000 deaths annually [1]. This poor prognosis is reflective of the advanced stage of many cases at presentation, with 61.8% of cases diagnosed in 2021 being Stage 3 or 4 in England [2]. As a strong predictor of survival, staging is an important part of the work-up of each case of ovarian cancer (Stage 4 vs. 1 having a risk ratio of 10.54) [3]. Surgical management for ovarian cancer consists of either primary debulking surgery (PDS) followed by platinum-based adjuvant chemotherapy, or neoadjuvant chemotherapy (NACT) followed by interval debulking surgery (IDS) [4]. As such, the diagnostic workload of tubo-ovarian and peritoneal cancer resection specimens is high, due to the large volume of distant-site tissue sampled (typically from the lymph nodes and omentum) for staging, along with the primary site and accompanying uterus.

This demand on histopathologist time occurs against the backdrop of a mismatch between the workload and the available workforce, with only 3% of UK departments reportedly having enough staff to meet clinical demand in 2017 [5]. This also translates to the global stage, where the mean number of pathologists per million population is 14, coupled with a huge disparity between developed and developing economies, e.g., 65 per million in the US compared to fewer than three (on average) per million in Africa [6]. Given the expected trends in population growth and ageing, it has been anticipated that there will be more than 35 million new cancer cases presenting worldwide in 2050, representing a 77% rise from 2022 figures [1]. Along with patient numbers increasing, the complexity of assessment per case is also rising, due to increased sampling and additional immunohistochemistry, genomics, and molecular testing [7]. One of the solutions suggested to alleviate the workload crisis is capital investment in the implementation of digital pathology, enabling efficiency as well as remote or flexible working [5]. A consequence of this digital expansion is the creation of large whole-slide image (WSI) data repositories which, when adequately curated, can form the basis for development of artificial intelligence (AI)-based solutions that have the potential to quantify more accurately than, and extract information that is beyond, human visual perception [8].

The field of AI in tubo-ovarian and peritoneal pathology research is swiftly expanding, having previously lagged behind that of other more common malignancies, a move from more traditional machine learning approaches to cutting-edge deep learning models [9]. The lag is, in part, due to the lack of substantial digital image repositories, with many studies relying on limited datasets, such as that offered by The Cancer Genome Atlas (TCGA) [10], which is only focused on primary tumour material. Much of the work conducted in relation to ovarian cancer to date has remained confined to the research environment, and no AI platforms have gained regulatory approval for clinical use [9]. More recently, models have utilised deep neural networks, with the focus of the research changing from tissue classification and stain quantification to morphological subtyping, breast cancer gene (BRCA) [11,12,13] and homologous recombination deficiency (HRD) status typing [14], serous tubal intraepithelial carcinoma (STIC) detection [15] and treatment response prediction [16,17,18]. Another multi-modal deep learning model has been developed that incorporates genomic data in addition to whole-slide images (WSIs) to predict the disease stage, although this was not designed to interpret WSIs of non-primary tumour-related tissue directly to provide a final pathological stage [19]. 

There appears to be growing international interest in integrating computer-aided diagnosis (CAD) and AI in diagnostic workflows, with a recent survey of 127 clinical institutions worldwide showing that 87.5% of respondents believed that synergy between pathologists and AI would complement current practice [20]. This survey also explored ideal applications for algorithms, with detection of metastasis in lymph nodes being the second most popular item on pathologists’ wish list after objective immunohistochemical scoring [20]. In this regard, compared to many other malignancies, the diagnostic workload of tubo-ovarian and peritoneal cancer resection specimens is high, due to the large volume of distant-site tissue sampled for staging (principally from the lymph nodes and omentum), along with the primary site and accompanying uterus. As such, detection of metastases in the context of ovarian cancer would be of great clinical interest. A number of endeavours have focused on this issue in terms of positive lymph node detection across a different range of malignancies, including melanoma [21], breast cancer [22,23,24,25,26], urothelial cancer [27], gastrointestinal cancer [28,29] and lung cancer [30]. However, to the best of the authors’ knowledge, an automated solution for the detection of metastases in the context of tubo-ovarian and peritoneal carcinomas is yet to be developed. The aim of this study was therefore to develop an AI model that could reliably identify the presence of metastatic carcinoma within the omentum and lymph nodes that accompanies the main tubo-ovarian or peritoneal carcinoma specimen, with a view to accelerating diagnostic turnaround times for histopathologists.

## 2. Materials and Methods

### 2.1. Ovarian Carcinoma Clinical and Pathology Data

Training and testing data were taken from The Bramall Ovarian Cancer Digital Pathology Repository, which comprises digital pathology WSIs and matched clinical data of 1000 tubo-ovarian and peritoneal cancer patients who underwent surgical management at Leeds Teaching Hospitals NHS Trust between 2008 and 2022. Patients were identified via searches for epithelial tubo-ovarian and peritoneal malignancies on the electronic health record (EHR) and pathology laboratory information management system (LIMS). Cases were included in the repository if they had one or more slides containing carcinoma, and covered a wide range of histopathology specimen types, including biopsies, resections, metastases and cytology. At least one pathologist (KEA/NMO) reviewed the glass slides for each case, blindly verified the diagnosis made clinically by a gynaecological pathologist and selected WSIs for inclusion. In the event of any diagnostic discrepancy, cases were removed from the study. Background benign tissue was also included within the repository to create a comparison cohort for studies involving malignancy detection. Selected H&E-stained glass slides made from formalin-fixed paraffin-embedded (FFPE) tissue were inspected for any mounting issues (e.g., air bubbles), which were corrected; these were then anonymised, cleaned with 70% ethanol, and digitised at 40× magnification on a Leica Aperio AT2 scanner. All resultant WSIs were quality-checked for scanning issues (e.g., poor stitching, being out of focus). Each WSI within the dataset was labelled at the slide level with the tissue type, malignancy status and morphological subtype of carcinoma, where present.

Two WSI subsets of omental and lymph node tissue from the main repository were identified, which either contained metastatic carcinoma or did not. Overall, this comprised training data from 715 WSIs (258 patients) and a hold-out independent test set of 140 WSIs (140 patients). Training and testing data included both PDS and IDS specimens, the latter of which can show morphological changes related to preoperative therapy. Whilst these can be considerable, such specimens have been found to benefit training data [31] and to make the model more clinically applicable. A breakdown of these cohorts is given in Table 1, and patient demographic and pathological details are given in Table 2. Morphological subtype imbalances were incorporated in the cohorts to reflect the prevalence observed at the population level. To prevent batch effects, we implemented careful data stratification, which maintained as consistent a distribution of morphological subtypes and sampling from different years (to mitigate possible staining differences) as possible across sets. Strict dataset boundaries for images and patches across training and hold-out test sets, as well as cautious and consistent file naming, were employed to prevent data leakage.

### 2.2. Whole-Slide Image Classification

Attention-based multiple-instance learning (ABMIL) [32] was used in this study for classification of WSIs as either benign tissue or containing a metastasis. Default procedures were drawn from the CLAM (clustering constrained-attention multiple-instance learning) method [33], as previously adjusted for ovarian cancer morphological subtyping [34]. Tissue was segmented from plain background using saturation thresholding. For patch extraction, 1024 × 1024 pixel non-overlapped areas of tissue were extracted at 40× magnification and downsampled to 256 × 256 pixels at 10× apparent magnification, which we found to be superior in previous classification-based work [35].

Patch features were extracted using the UNI foundation model, a self-supervised model that was pre-trained on over 100 million images from H&E-stained WSIs of 20 major tissue types [36]. This particular feature extractor was chosen following a rigorous comparison of fourteen foundation models and three ImageNet-pre-trained encoders for the task of ovarian cancer morphological classification, where the UNI model was one of the best-performing models, whilst also proving efficient, with a fast running time [34]. 

**Figure 1:**
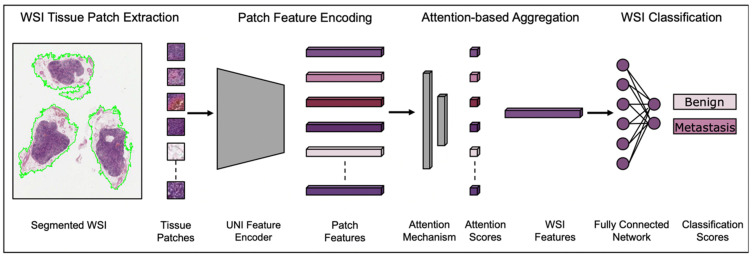
Classification pipeline. The ABMIL classifier for the assessment of tissue accompanying the main ovarian cancer specimen for the presence of metastases—in this example, a lymph node. Adapted from [34].

The patch features were used to train an ABMIL classifier by passing them through a trainable attention layer, where each was assigned an attention score between 0 and 1 that represented the relative importance of that patch in the end classification. WSI-level features were then produced from the attention-weighted average of the patch features, and then classified through the fully connected neural network with one output node per class. The output was passed through the softmax function to generate the classification probabilities for each of the two classes (either benign or metastasis), with the maximum taken as the prediction. The full process is represented in Figure 1. Hyperparameters for this task were determined based on a previous study by our group, where ABMIL was tuned using ResNet50 features for another classification task of morphological subtyping with a different subset of the same repository [35]. Dropout, weight decay and early stopping were employed to prevent model memorisation and overfitting to specific training patterns.

### 2.3. Model Evaluation

The model was evaluated using accuracy, balanced accuracy, area under the receiver operating characteristic curve (AUROC) and F1 score. These metrics were chosen to assess different aspects of classification performance, with AUROC giving a holistic, albeit imbalanced, interpretation of discriminative power. F1 provided a balanced metric of prediction at a set threshold. Finally, balanced accuracy offered a more realistic assessment of clinical performance. Training was centred on the use of stratified five-fold cross-validation (split 60-20-20 training–validation–testing at the case level to avoid data leakage and prevent model memorisation). Predictions of the five cross-validation models were averaged, generating an ensembled classification in our hold-out testing. All results are reported using the mean and 95% confidence interval (CI) from 10,000 iterations of bootstrapping.

## 3. Results

### 3.1. Lymph Node Metastasis Evaluation

**Figure 2:**
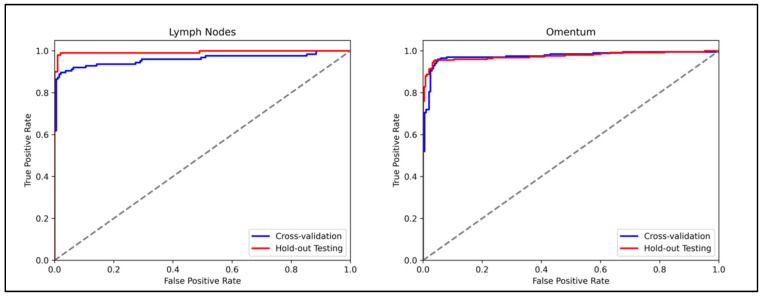
Receiver-operating characteristic curves for the model for each of the two cohorts.

The model to detect metastatic carcinoma within lymph nodes achieved an AUROC of 0.959 (95% CI, 0.929–0.983) in cross-validation, and an AUROC of 0.998 (95% CI, 0.985–1.0) on the hold-out test set. The ROC curves are presented in Figure 2. Better performance was seen across all metrics within the hold-out test set compared to the internal training cross-validation (Table 3), with all 20 of the test set WSIs being correctly classified when the five-fold cross-validation models were ensembled. This gave a precision, sensitivity, specificity and F1 score for both the classifications of benign and malignant of 1.000.

### 3.2. Omentum Metastasis Evaluation

**Figure 3:**
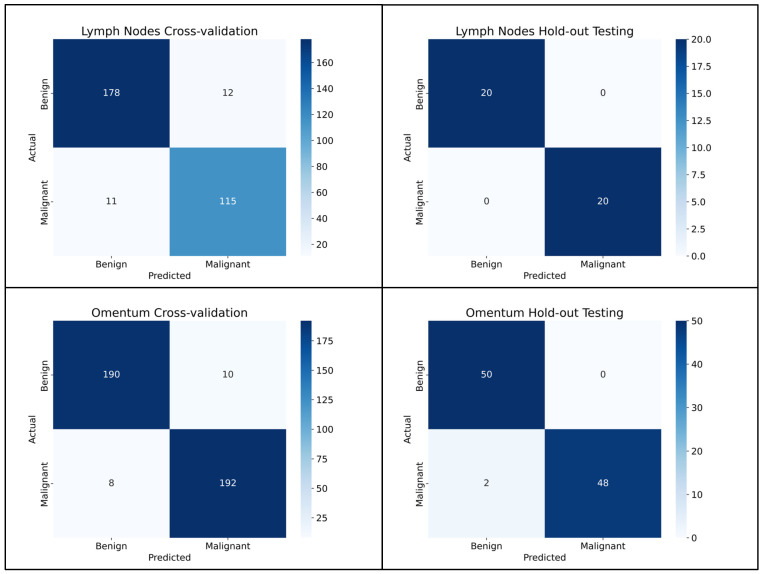
Confusion matrices for the cross-validation and hold-out testing for the lymph node and omentum cohorts.

The model to detect metastatic carcinoma within omental tissue reached an AUROC of 0.975 (95% CI, 0.958–0.989) in training cross-validation, and an AUROC of 0.963 (95% CI, 0.911–0.999) on the hold-out test set. The ROC curves for both the training and test sets are presented in Figure 2. This superior performance in the training cross-validation was only noted when using AUROC as a metric. Performance was better in the hold-out test set for accuracy, balanced accuracy and F1 than it was in training cross-validation. The results for all metrics are shown in Table 3. All benign WSIs were correctly classified in the hold-out test set when the five-fold cross-validation was averaged, with two of the malignant WSIs misclassified as benign. A summary is given in the confusion matrices in Figure 3. For the benign classification, the precision was 0.962, the sensitivity was 1.000, the specificity was 0.960 and the F1 was 0.980. For the classification of WSIs containing metastasis, the precision was 1.000, the sensitivity was 0.960, the specificity was 1.000 and the F1 was 0.980.

### 3.3. Attention Heatmaps

**Figure 4:**
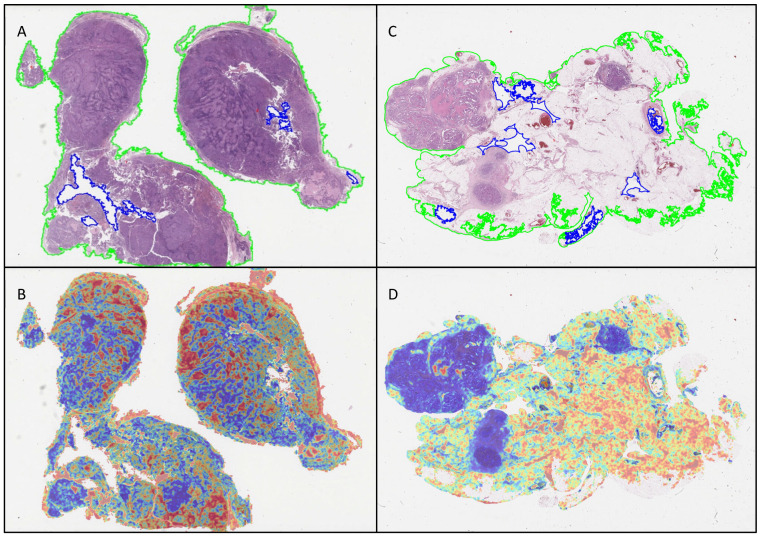
Heatmaps highlighting model attention in comparison to the segmented WSIs for (**A**,**B**) lymph node and (**C**,**D**) omental tissue containing metastatic carcinoma.

ABMIL attention heatmaps for each WSI were generated, based on 256 × 256 pixel patches with 50% overlap at 10× apparent magnification, to enable qualitative assessment of the behaviour of the model. Examples of heatmaps produced by the model are shown in Figure 4. A sample of these was interpreted qualitatively by two pathologists (KEA and NMO) in relation to the morphological features seen in the WSIs. In general, the heatmaps for the lymph node cohort showed that there was more attention paid to background lymph node architecture than to the metastatic carcinoma cells and immediately adjacent tissue. Similarly, the heatmaps for the omentum cohort showed that more attention was paid to areas of fibroadipose tissue and lymphovascular spaces than to metastatic carcinoma.

## 4. Discussion

Internationally, histopathologists have expressed an interest in using AI as an adjunct to assist in the reporting of accompanying material to the primary tumour specimen (such as lymph nodes) in the context of staging [20]. While metastatic lymph node and omental involvement is key to ovarian cancer staging, treatment planning and prognosis, the assessment of these tissues can be laborious and time-consuming. This study sought to develop and validate an AI model that could assist pathologists by identifying whether a WSI contains an ovarian carcinoma metastasis. 

To the best of the authors’ knowledge, this is the only study specifically aimed at detecting ovarian cancer metastases. With a balanced accuracy of 100% and 98.0% seen in the lymph node and omentum test cohorts, respectively, this model shows great promise as an adjunct to pathologist review of ovarian cancer primary or interval debulking surgery. Strikingly, classification performance was not just maintained, but shown to be higher, in the hold-out test set (of WSIs that the model was not exposed to during training) across all performance metrics in the lymph node cohort, and in all but AUROC in the omentum cohort. This highlights the reproducibility, robustness and adaptability of the model when deployed for new cases within our institution. In a clinical setting, the potential of this platform could be exploited in the form of an integrated application in digital pathology workflows, where it could be utilised as a tool for screening and prioritisation of WSIs prior to a pathologist’s review. Such a workflow could comprise slide scanning, and then flagging of omentum or lymph node WSIs from the macroscopic description. These could then be automatically analysed by either the lymph node- or omentum-tuned model (ideally with a measure of confidence of the prediction) and this analysis made available prior to pathologist review and reporting. 

As outlined earlier, analogous models have been developed for the detection of nodal disease in other solid malignancies. The most notable work has been in breast pathology, where there is a plethora of WSI training data, including the rigorously annotated CAMELYON16 and CAMELYON17 datasets [23]. Using these data, Google researchers developed a deep learning algorithm named LYNA, which was able to achieve an AUC of 0.994 in detecting metastatic breast carcinoma, outperformed pathologists under time constraints, and identified slides ground-truth-labelled as benign to actually contain micrometastases, highlighting how such models can increase diagnostic accuracy [37]. Another diagnostic AI platform, VisioPharm’s Integrator System, has been designed to detect lymph node metastases within a clinical digital workflow. This was able to detect all metastases within both the sentinel (234 lymph nodes) and non-sentinel (256 lymph nodes) validation cohorts, with 100% sensitivity and 41.5% specificity within the sentinel node cohort specifically [24]. Other recent models have also explored the benefits of using AI purely as an adjunct to pathologist opinion. For example, Ratamero et al. showed a significant improvements in the sensitivity of pathologists’ assessments of breast sentinel lymph node metastases from 74.5% to 93.5%, translating into an efficiency gain of 55% in reading time, by highlighting areas suspicious for metastasis [26]. 

**Figure 5:**
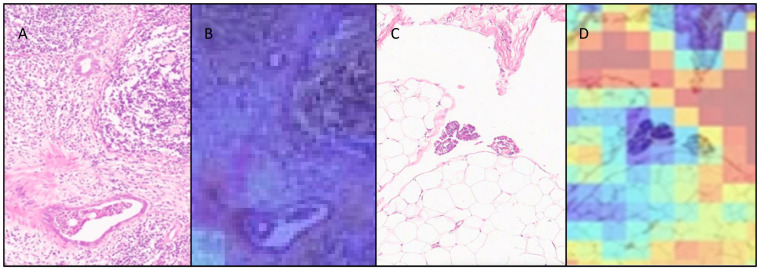
Comparison of areas within H&E WSIs and heatmaps (with a gradient of high to low attention, from red to blue) where metastases were misclassified as benign within individual folds of the ensembled model. (**A**) An area of a WSI containing metastatic carcinoma in a lymph node; (**B**) an overlaid heatmap for (**A**) showing low attention over metastatic carcinoma; (**C**) an area of a WSI of an omentum containing metastatic carcinoma; (**D**) an overlaid heatmap for (**C**) showing lower attention over metastatic carcinoma.

Across the five sub-models from the five cross-validation folds, only three test set lymph node WSIs were misclassified (one false negative and two false positives), with each only misclassified by one of the single-fold models, such that they were classified correctly overall by the ensembled model predictions. Each of these WSIs was qualitatively assessed by two pathologists (KEA, NMO). Interestingly, benign lymph nodes misclassified as being malignant displayed a range of patterns seen in reactive hyperplasia, including sinus histiocytosis, follicular hyperplasia and paracortical hyperplasia. Anecdotally, while benign, these are changes to lymph node architecture that commonly would prompt a pathologist to conduct a more in-depth assessment of a WSI. By contrast, the heat map for the malignant lymph node misclassified as being benign in only one fold showed that the small focus of malignant cells seen lining an intracapsular lymphovascular channel were in an area of low attention (Figure 5). 

Assessment of the individual folds in the omentum test set revealed that eight WSIs were misclassified (four false positives and four false negatives). One of the false negative WSIs was misclassified in only one fold, and another was classified with low probability in two folds, with the ensembled classification being correct in these cases. Only two WSIs containing metastases were misclassified across all five folds with high probability scores, and therefore were incorrectly classified overall. A qualitative review of the WSIs classified as false negative revealed that in both instances, the metastases were inconspicuous (maximum 100 microns in diameter), with low- and intermediate-grade features that might be misinterpreted by a human pathologist as reactive mesothelium. One was a grade 2 mucinous carcinoma unaccompanied by extracellular mucin; the other was a low-grade serous carcinoma associated with psammoma bodies. On the heatmaps, the areas with metastases showed low attention. Three of the false positives were only misclassified in two folds, and one in three folds, with low probability; therefore, the ensembled classification was correct for all. Interestingly, benign WSIs that were misclassified as being malignant shared a common pattern: all exhibited a prominent mixed inflammatory cell infiltrate, as well as featuring a mesothelium displaying reactive features. The heatmaps for these cases showed that high attention was concentrated on areas of inflammation, as well as on erythrocytes within ectatic blood vessels. These findings may indicate that the training set did not contain sufficient examples of these omental features, although, anecdotally, they would also be findings that might attract a pathologist to conduct a high-power assessment. 

Overall, the attention heatmaps have highlighted that the model showed high attention on both the lymph node and omentum WSIs in areas where there were no malignant cells. This could indicate that the model detects background changes in morphology that are associated with malignancy, such as alterations in normal lymph node architecture, inflammation or stromal changes that are part of the tumour microenvironment. However, as it could also indicate insufficient training or bias, our future work will incorporate analysis of the heatmap visualisations to further investigate the model’s focus in making predictions.

One of the notable benefits of this study is that it did not require labour-intensive manual annotation, with labelling at the slide level only. It also exclusively relied on H&E-stained tissue, rather than using immunohistochemical staining to highlight malignant cells. In a clinical context, this could potentially minimise the amount of laboratory resources, workforce and costs required, and could thus prove useful in developing economies. Moreover, the model was deployed on a desktop computer with increased memory and a dedicated graphics card, rather than on a high-performance computing platform, and, as such, could be easily incorporated into most healthcare infrastructures. Finally, in order to develop this model, we had to assemble The Bramall Ovarian Cancer Digital Pathology Repository, which is currently the only ovarian dataset suitable for the development of metastasis detection models globally. Its reach also has the potential to expand this work to analysing tumour deposits in other metastatic sites (e.g., gastrointestinal serosa and mesentery, uterus, bladder, peritoneum), as well as supporting the inception of other ovarian cancer diagnostic and prognostic models. 

There are several limitations to the study. In the application of the model to the hold-out test-sets, there were a very small number of misclassifications observed in the individual folds, but this was mitigated by using ensembled predictions. The hold-out test sets were also relatively small, particularly in the lymph node cohort, and therefore, a level of uncertainty should be assumed. There is also a possibility of overfitting in the evaluation of performance, not just in the training five-fold cross-validation, but also on our hold-out test set, as our training and test data originated from a single centre; thus, the model may have inadvertently learnt, and become reliant on, centre-specific features. These could include staining protocols, scanner characteristics and sample preparation techniques, and until we apply the model to an external dataset, we will not be able to determine the extent of any overestimation of the model’s true performance. In future expansions of the study, we will also explore stain normalisation techniques and colour augmentation to reduce the impact of scanner- or protocol-specific visual features, further preventing batch effects. Furthermore, we will investigate the use of aggressive data augmentation techniques (e.g., rotation, flipping, blurring, etc.) that could encourage the model to learn robust, generalisable features. The hyperparameters for this study were taken from our previous investigation that employed the ResNet50 feature extractor for the task of ovarian carcinoma morphological subtyping [35]. In future iterations of this study, tuning these could deliver a better model performance and make it more computationally efficient. Given that the misclassifications in the omentum test cohort were false negatives, it would be advisable that all WSIs undergo pathologist review prior to sign-out. These interpretations also had high predicted probability values associated with the incorrect classification within the model, meaning that these scores may not offer a reliable measure of algorithm confidence as part of the clinical workflow. Moreover, in a clinical setting, the algorithm would not be able to establish a difference between a lymph node metastasis and a tumour deposit, and thus would still rely on a pathologist’s assessment. 

In terms of future directions, the next appropriate step for this model would be to apply it to an external validation set (i.e., collected outwith our institution), which would enable us to assess its generalisability. Unfortunately, there is a paucity of open-source digital pathology data in the field of ovarian cancer, which limits opportunities for external validations of both this and other research [9]. The datasets that are currently available to investigators, including those supporting the TransCanadian Study [38], the OCEAN AI Challenge [39] and TCGA-OV [10], either do not specify the exact anatomical location of the tissue available, or comprise tumour-containing tissue from either the adnexae or omentum, with a background of fibroadipose tissue. The ideal progression would be to extend the present study to incorporate larger, more diverse training and testing sets, including data from multiple centres with WSIs generated on a variety of different scanners from H&E-stained tissues prepared in different laboratories. In addition, both the training and testing cohorts used in this study could also be expanded to include an increased representation of rarer morphological subtypes, in order to increase the real-world applicability. In the design of this study, we chose not to incorporate more detailed pathologist WSI annotation, as this would not have addressed its principal aim. Nevertheless, we plan to incorporate such orthogonal validations in future studies to compare the attention overlap of the model. Further strategies we consider employing to improve specificity include integrating a model for cell-level segmentation as an auxiliary input, or using uncertainty modelling to identify ambiguous regions, adjust the decision threshold or trigger an additional pathologist review. Metastases of all different sizes were included in both the lymph node and omental dataset in this study. In future work, the size of the metastases within the WSIs would also be assessed. Whilst this currently does not form part of reporting in ovarian cancer for either the omentum or extraperitoneal lymph nodes, it is part of FIGO staging for retroperitoneal lymph nodes, with metastases measuring up to 10 mm being regarded as IIIA1(i) and those > 10 mm being regarded as IIIA1(ii) [40]. The American Joint Committee on Cancer (AJCC)’s classification of lymph node metastases has been taken up for other malignancies, such as breast, endometrial and cervical malignancies. Given that the current FIGO staging for ovarian cancer was developed in 2014, it could be assumed that the next iteration may utilise the AJCC classification, and therefore, it may be beneficial to use the categories of isolated tumour cells (diameter ≤ 0.2 mm), micrometastases (>0.2 but ≤2.0 mm) or macrometastases (>2.0 mm) in future studies [41].

While our model demonstrated excellent accuracy on this single-centre dataset in detecting ovarian cancer nodal and omental metastases, we acknowledge the need for a balanced evaluation of its clinical implications, particularly regarding false negatives and false positives. False negatives, or missed metastases, may delay appropriate treatment escalation through inaccurate staging, while false positives could lead to overtreatment. However, in the context of high-volume pathology workflows, the time-saving potential of automated pre-screening tools remains significant. By triaging negative cases and prioritising slides likely to contain metastases, the model could reduce diagnostic fatigue and improve overall pathologist throughput. To support its safe integration into clinical practice, our future work will also assess the model’s performance in a human-in-the-loop setting, including re-review of discordant cases and establishing the potential downstream impact on patient management. A risk–benefit analysis considering these factors will be key to validating the utility of the model beyond simply delivering its standalone performance metrics.

## 5. Conclusions

This study illustrates the potential of AI solutions as a diagnostic adjunct informing prognostic performance as part of pathological staging of tubo-ovarian and peritoneal malignancies. The performance levels achieved herein—if proven to be sustained on independent datasets—are sufficiently promising to be considered useful in the context of routine diagnostic workflows. The tangible benefits of automated lymph node and omental tumour deposit identification would include accelerated turnaround times, streamlined workflows and potential reductions in diagnostic error. The utility of this algorithm could come into its own as a pre-screening tool deployed prior to histopathologist review which could benefit both healthcare services and, ultimately, patients.

## Figures and Tables

**Table 1:** Breakdown of lymph node and omentum training and hold-out testing sets.

	LYMPH NODES		OMENTUM	
	Training WSIs (Patients)	Hold-Out Testing WSIs (Patients)	Training WSIs (Patients)	Hold-Out Testing WSIs (Patients)
**Benign**	189 (48)	20 (20)	200 (57)	50 (50)
**Malignant**	126 (65)	20 (20)	200 (106)	50 (50)
**Overall**	315 (113)	40 (40)	400 (161)	100 (100)

**Table 2:** Patient demographic and pathological details.

Characteristic			Lymph Node Cohort (*n* = 113)	Omentum Cohort (*n* = 161)
**Age**	Mean (SD)		59.8 (12.5)	61.2 (13.1)
	Median (IQR)		60.0 (51.5–68.5)	62.0 (52.0–71.0)
FIGO * stage	1		30	37
	2		6	9
	3		61	94
	4		16	21
**S** **urgery type**	PDS		85	122
	IDS		27	39
**Morphological subtype and grade**	High-grade serous carcinoma		66	93
	Low-grade serous carcinoma		3	3
	Clear-cell carcinoma		11	16
	Endometrioid carcinoma	G1	5	7
		G2	6	7
		G3	7	10
	Mucinous carcinoma	G1	2	4
		G2	2	5
		G3	0	0
		UG	3	4
	Mixed	HG	5	6
		LG	0	0
	Carcinosarcoma		4	6

* International Federation of Gynecology and Obstetrics (FIGO).

**Table 3:** Results of classification of lymph nodes and omentum for presence of carcinoma metastases from training 5-fold cross validation and hold-out testing across multiple metrics.

	LYMPH NODES		OMENTUM	
METRIC	Cross-Validation (95% CI)	Hold-Out Testing (95% CI)	Cross-Validation (95% CI)	Hold-Out Testing (95% CI)
**AUROC**	0.959 (0.929–0.983)	0.998 (0.985–1.0)	0.975 (0.958–0.989)	0.963 (0.911–0.999)
**Accuracy**	92.7% (89.6–95.3%)	100.0% (100.0–100.0%)	95.4% (93.5–97.5%)	98.0 (95.0–100.0%)
**Balanced Accuracy**	92.4% (89.2–95.3%)	100.0% (100.0–100.0%)	95.5% (93.4–97.5%)	98.0% (94.8–100.0%)
**F1**	0.908 (0.868–0.943)	1.0 (1.0–1.0)	0.955 (0.933–0.975)	0.979 (0.945–1.0)

## Data Availability

Approval for these data to be shared outside of the research group is awaited. Please contact the authors for information on collaboration. All the code used in this research is available at https://github.com/kea-llen/Ovarian_Features (accessed on 13 February 2025).

## Abbreviations

The following abbreviations are used in this manuscript:

AJCC	American Joint Committee on Cancer
AUROC	Area under the receiver operating characteristic curve
AI	Artificial intelligence
ABMIL	Attention-based multiple-instance learning
BRCA	Breast cancer gene
CLAM	Clustering constrained-attention multiple-instance learning
CAD	Computer-aided diagnosis
CI	Confidence intervals
EHR	Electronic health record
FFPE	Formalin-fixed paraffin-embedded
HRD	Homologous recombination deficiency
FIGO	International Federation of Gynecology and Obstetrics
IDS	Interval debulking surgery
LIMS	Laboratory information management systems
NACT	Neoadjuvant chemotherapy
PDS	Primary debulking surgery
STIC	Serous tubal intraepithelial carcinoma
TCGA	The Cancer Genome Atlas
WSI	Whole-slide image
